# Lightsheet fluorescence lifetime imaging microscopy with wide‐field time‐correlated single photon counting

**DOI:** 10.1002/jbio.201960099

**Published:** 2019-11-25

**Authors:** Liisa M. Hirvonen, Jakub Nedbal, Norah Almutairi, Thomas A. Phillips, Wolfgang Becker, Thomas Conneely, James Milnes, Susan Cox, Stephen Stürzenbaum, Klaus Suhling

**Affiliations:** ^1^ Randall Centre for Cell and Molecular Biophysics King's College London London UK; ^2^ Department of Physics King's College London London UK; ^3^ School of Population Health & Environmental Sciences, Faculty of Life Sciences & Medicine King's College London London UK; ^4^ Becker & Hickl GmbH Berlin Germany; ^5^ Photek Ltd. Saint Leonards‐on‐Sea UK

**Keywords:** fluorescence lifetime imaging (FLIM), lightsheet microscopy, microchannel plate (MCP), SPIM, time‐correlated single photon counting (TCSPC)

## Abstract

We report on wide‐field time‐correlated single photon counting (TCSPC)‐based fluorescence lifetime imaging microscopy (FLIM) with lightsheet illumination. A pulsed diode laser is used for excitation, and a crossed delay line anode image intensifier, effectively a single‐photon sensitive camera, is used to record the position and arrival time of the photons with picosecond time resolution, combining low illumination intensity of microwatts with wide‐field data collection. We pair this detector with the lightsheet illumination technique, and apply it to 3D FLIM imaging of dye gradients in human cancer cell spheroids, and *C. elegans*.
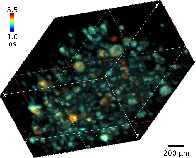

## Graphical abstract

We combine fluorescence lifetime imaging (FLIM) with lightsheet illumination microscopy for 3D imaging of human cancer cell spheroids and living *C. elegans*. The wide‐field TCSPC method used for FLIM detection has many advantages including extremely low illumination intensity, good signal‐to‐noise ratio and insensitivity to intensity fluctuations and photobleaching, while lightsheet illumination provides optical sectioning, making this technique ideal for 3D FLIM imaging of living organelles.

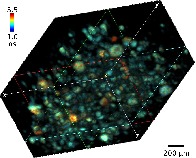

## 1 INTRODUCTION

The observation of cells and organelles in their native 3D environment is becoming increasingly important in biological research 1. Fluorescence microscopy provides a way to do this, as it is a minimally invasive, non‐destructive and non‐ionising tool which can be used to study living cells and tissues with negligible cytotoxicity, so that dynamics and function can be observed and quantified. No other method can study molecules in living cells with anything remotely approaching its combination of spatial resolution, selectivity, sensitivity and dynamics.

In a conventional fluorescence microscope, the sample is typically placed on a glass coverslip, and imaging is restricted to a region relatively close to the coverslip, as resolution and contrast decrease rapidly with imaging depth, which can be problematic for larger samples. Also bleaching and phototoxicity can become issues in 3D imaging due to prolonged illumination of the whole sample volume. Multiphoton excitation microscopy helps to address these issues, and can image relatively large samples when using a mesolens 2, 3. This is a scanning approach where the image is acquired pixel by pixel. To acquire the whole field of view in a single exposure using a camera while still addressing the photobleaching and depth issue, lightsheet illumination can be used.

**Figure 1:**
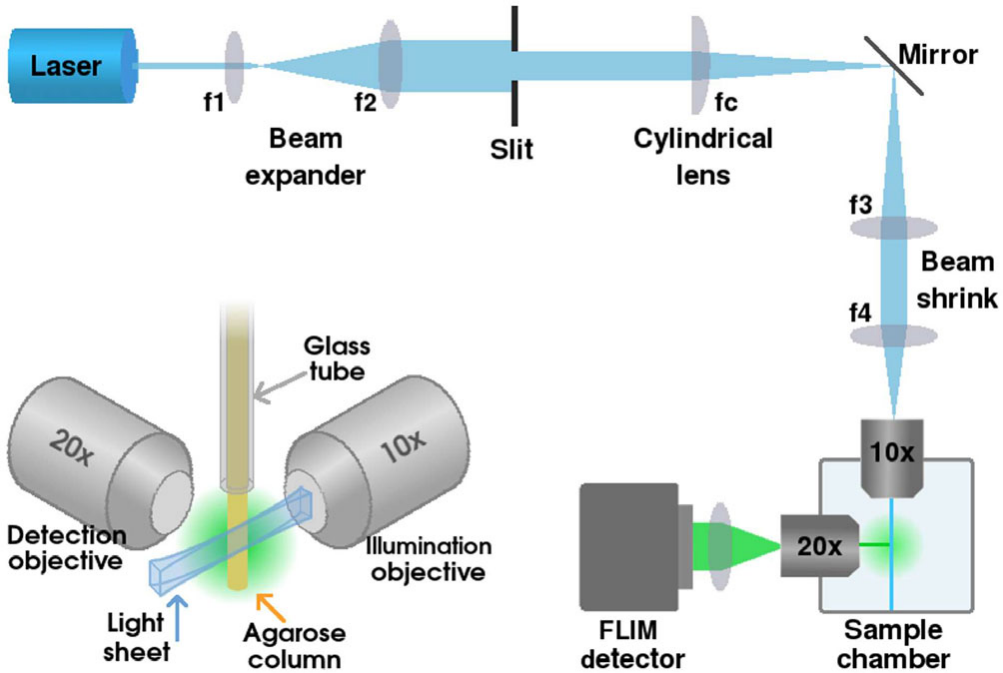
Schematic diagram of the lightsheet FLIM microscope setup. Inset: The sample is mounted in an agarose column and placed on the intersecting focal planes of the illumination and detection objectives

In the past 10 years, developments in lightsheet illumination techniques have advanced biological 3D microscopy enormously. Also called single/selective plane illumination microscopy (SPIM) and named Nature Methods “Method of the Year 2014” 4, lightsheet microscopy provides inherent optical sectioning and reduces photobleaching outside the focal plane. While the sideways illumination technique is not new – it was first introduced in 1902 by Zsigmondy to look at scattered light from colloids in solutions, for which he won the Chemistry Nobel prize in 1925 5 – its recent re‐discovery and utilisation in fluorescence microscopy has become a powerful and popular technique. It allows image acquisition with depth resolution provided by the illumination lightsheet, without bleaching the parts of the sample that are not imaged. It is typically applied to biological imaging of larger multicellular organelles, such as cell clusters, tissues or embryos, with low magnification and a large field of view, but it also has applications in single cell imaging. Indeed, lightsheet microscopy has been demonstrated in single molecule scale and combined with superresolution microscopy 6. It can be performed over long periods of time with very low phototoxicity. Typically, the sample is mounted in a highly viscous medium such as agarose gel, placed in a small transparent tube and mounted in front of the camera (see Figure 1 inset). It is possible to rotate the sample and acquire three‐dimensional stacks from various angles. There are many different practical implementations of lightsheet microscopy, each with their benefits and drawbacks and suitability for a specific application 7. As it is not based on point‐scanning an excitation beam, it usually requires a camera to acquire the image.

Fluorescence lifetime imaging microscopy (FLIM) provides contrast according to the time the fluorophore spends in the excited state. This often depends on the microenvironment of the probe, such as oxygen or ion concentration, pH, viscosity, temperature, or proximity of other fluorophores. A great advantage of combining FLIM with lightsheet microscopy is that it enables functional imaging, i.e. it can go beyond the structural and morphological information that is obtained from standard implementations of lightsheet microscopy. There are some reports on combining lightsheet illumination with FLIM, usually either with frequency domain 8, 9 or time‐gated FLIM 10, 11 approaches 12, 13, 14, 15, but only one report, to the best of our knowledge, on the use of time‐correlated single photon counting (TCSPC) for lightsheet FLIM microscopy 16. The authors use a prototype image intensifier with capacitive division imaging readout technique, which is based on charge division to determine the photon position and arrival timing 17. They image fluorescent beads and a calcium sensitive dye in buffer solution of varying calcium ion concentrations, and drosophila larvae.

TCSPC has been reported to have the best signal‐to‐noise ratio of the standard time‐resolved imaging methods. It is independent of excitation intensity variations, and its other key advantages stemming from its digital nature include single photon sensitivity, a high dynamic range, linearity, well‐defined Poisson statistics and easy visualisation of photon arrival time data 18, 19. FLIM is often implemented in a scanning system, but there are also various methods to perform wide‐field TCSPC 20. Traditionally, microchannel plate (MCP) detectors have been used for timing photon arrival with precision of few tens of picoseconds 21. To also record the position, different read‐out anode architectures have been developed 22, 23, 24, 25. Quadrant anodes have been used for long‐term wide‐field TCSPC FLIM imaging with low excitation intensity 26, 27, 28. In a delay line anode detector, the problem of determining the position of the photon event is converted into a timing problem. We have previously shown that this type of detector can be read out with standard TCSPC timing electronics, such as timing boards that are routinely used for fluorescence decay measurements, for example in combination with scanning FLIM 29. We have also employed this detector for total internal reflection FLIM, a method that typically requires a camera 30.

The trend towards higher resolution and ever thinner optical sections and the limited photon budget available from fluorophores make photon counting the method of choice for lightsheet FLIM. In this work we have combined wide‐field TCSPC FLIM with lightsheet illumination using two orthogonal objectives. For TCSPC detection, we have combined a microchannel plate (MCP)‐based detector with a delay line position sensitive anode and fast electronics 29, 30. This detection system allows wide‐field FLIM with picosecond time resolution and extremely low illumination intensity. We employ this detector on lightsheet illumination microscope, and apply this system to FLIM of human cancer cell spheroids and *C. elegans*.

## 2 METHOD

### 2.1 Microscope setup

The lightsheet microscope setup is based on the openSPIM 31 setup geometry, see Figure 1. A Horiba DeltaDiode picosecond diode laser with 485 nm head (DD‐485L) was used for excitation at 10 MHz repetition rate. A 475/28 filter was inserted in front of the laser, and neutral density filters were used in the excitation path to reduce the illumination intensity. The beam was expanded with two lenses (*f*_1_ = 50 mm, *f*_2_ = 100 mm), the lightsheet was created with a slit and a cylindrical lens (*f*_*c*_ = 50 mm), and the beam diameter was then decreased with another two lenses (*f*_3_ = 100 mm, *f*_4_ = 50 mm). The lightsheet was focused onto the sample with a 10× NA0.3 water immersion objective (UMPLFLN10XW, Olympus), mounted onto the side of a water‐filled sample chamber. The fluorescence from the sample was collected with a 20× NA0.5 water immersion objective (UMPLFLN20XW, Olympus), mounted onto another side the sample chamber perpendicular to the excitation objective such that their focal planes intersect. A 515 nm long‐pass emission filter (FITC‐LP01‐Clin‐25, Semrock) was placed in the emission path, and the image was focused onto the detector with a tube lens (TTL200‐A, Thorlabs).

For imaging, the sample chamber was filled with deionised water (or M9 buffer for *C. elegans*). The samples were suspended in agarose (RC‐122, G‐Biosciences) and mounted into 1 mm diameter glass capillaries (701904, Brand). The capillaries were mounted on a micrometre stage on top of the sample chamber such that the end of the capillary was slightly above the focal plane of the objectives, and the agarose was then pushed out of the capillary into the beam path where the focal planes of the objectives intersect (see Figure 1 inset).

### 2.2 FLIM detector

The delay line detector used in this work has been described in detail elsewhere 29, 30. Briefly, the 40 mm double MCP detector (Photek, UK) was combined with a 4‐channel delay line read‐out anode structure (DLD40, Roentdek, Germany) located outside the tube and coupled capacitively to a resistive anode inside the tube using image charge technique 32, 33. The output signal from the MCP before the anode (time channel) was input to a SPC‐150 TCSPC module (Becker & Hickl, Germany), and the timing done in the conventional way 18. The delay line output signals (*X*_0_, *X*_1_, *Y*_0_ and *Y*_1_) were connected to constant fraction discriminators (CFDs) and amplifiers (Roentdek, Germany) that were optimised for slow pulse rise times as detailed in Reference 29 and then connected to two SPC‐150 TCSPC modules (Becker & Hickl, Germany), one for *X* and one for *Y*.

*X*_1_ and *Y*_1_ signals were connected to the signal inputs, and the stop signals were obtained from *X*_0_ and *Y*_0_ after passing through a 10 meter delay cable. This ensures that the *X*_1_ and *Y*_1_ signals always arrive first and start the TCSPC boards' time‐to‐amplitude converters, and the *X*_0_ and *Y*_0_ signals stop them. This setup thus measures the propagation time difference of the signal along the delay line, and therefore the photon event location. Data was collected with SPCM instrument control software (Becker & Hickl, Germany).

### 2.3 Data processing

Data was recorded in first‐in first‐out (FIFO, Parameter‐Tag) mode, where each SPC‐150 card writes a data file with two time stamps for each photon: microtime (time since the last excitation pulse) and macrotime (time since the start of the experiment). The three output files were combined by finding events that were found in the same macrotime window, corresponding to the time between laser pulses, in all three input files. The result file then only has photon events which were detected in all three (*x*, *y* and *t*) channels 18. Due to the broad pulse height distribution of the MCP and ringing in the pulse shape caused by the delay line structure, not all events are detected in all three channels. The 100 ns detection window was divided into 512 bins (pixels) for the *x*, *y* and *t* channels, thus yielding a calibration of 0.1953 ns/channel.

For the creation of the lifetime images, the photons were placed into an *xyt* data cube, and the data written into an “.ics” image file. The fluorescence decay in each pixel of the image was fitted with a monoexponential or biexponential function using Tri2 34 or SLIM Curve 35 software, and the lifetime encoded in a pseudocolor scale (blue for short lifetimes and red for long lifetimes). The lifetime and intensity images were combined by weighting the lifetime image pixel brightness values by the fluorescence intensity image. Average lifetimes for biexponential fits were obtained from the intensity‐averaged fluorescence lifetime $\tilde{\tau }\;=\;\frac{\left(\alpha_{1},{\tau_{1}}^{2},+,\alpha_{2},{\tau_{2}}^{2}\right)}{\left(\alpha_{1},\tau_{1},+,\alpha_{2},\tau_{2}\right)}$ 36. For volume sweep imaging, lifetime *τ* was calculated using the method of moments 37, 38. The first moment of the photon distribution is defined as $M_{1}=\frac{\sum N_{i}t_{i}}{N}$, where *t*_*i*_ is the time of the time channel *i*, *N*_*i*_ is the number of photons in time channel *i*, and *N* is the total number of time channels. *τ* was then obtained from the difference between the fluorescence decay and IRF: *τ*_M1fluo_ − *τ*_M1IRF_.

### 2.4 Sample preparation

#### 2.4.1 Beads

Two different types of fluorescent bead samples were used for testing the setup. For the first sample, two types of green fluorescent beads with 10 μm diameter (G1000, Thermo Fisher Scientific and 94050, Sigma‐Aldrich) were mixed and suspended in agarose (A9414, Sigma Aldrich). For the second sample, fluorescent 1 μm beads (F8823, Invitrogen) and quantum dots (Lumidot CdSe/ZnS, 694649, Sigma) were mixed such that they spontaneously formed aggregates in the agarose.

#### 2.4.2 Cancer cell spheroids

MCF‐7 cells (ATCC HTB‐22) were cultured in 37°C, 5% CO_2_ in EMEM supplemented with 10% FBS, 1% penicillin/streptomycin, and 1% l‐glutamine. For MCF‐7 cell line with stable GFP‐lifeact expression, lentivirus encoding lifeact‐GFP 39 (a gift from R. Wedlich‐Soldner, Max Planck Institute of Biochemistry, Martinsried, Germany) was packaged in HEK293T cells by transient transfection, and the supernatants containing lentivirus harvested after 48 hours. The MCF‐7 cells were then incubated with lentiviral supernatants for 24 hours, and sorted for optimal GFP expression. Spheroids were prepared by the hanging drop method adapted from Reference 40. For the methylcellulose (MC) stock 6 g was dissolved in 250 mL DMEM and stirred for 20 minutes at RT, the solution was then stirred overnight at 4°C with additional 250 mL of DMEM and clarified by centrifuging. 150 000 cells were suspended in 3 mL of 25% MC in growth medium, and 30 μL drops were deposited onto the lid of a square petri dish. The lid was then inverted over the dish bottom containing PBS, and the hanging drops incubated at 37°C, 5% CO_2_ to allow spheroids to form. After 24 hours the spheroids were fixed in 4% formaldehyde for 2 hours and washed thoroughly. For spheroid surface labelling Alexa488‐conjugated Tom20 antibody (sc‐17764‐AF488, Santa Cruz Biotechnology) was diluted 1:25 in PBS and the spheroids incubated for 1 hour. The samples were washed thoroughly, mounted into agarose and imaged immediately.

#### 2.4.3 *C. elegans*

The standard method by Brenner 41 was used to culture a transgenic *Caenorhabditis elegans* strain which expresses *Pmtl*‐1::GFP only upon exposure to cadmium. Synchronised (L1 stage) larva of the transgenic worms were grown on Nematode Growth Media (NGM) and fed ad libitum with *E. coli* OP50 in the presence or absence of 60 μM cadmium chloride (Sigma‐Aldrich 99% A.C.S – CdCl_2_). The nematodes were collected after 27 hours (L3 stage), then washed multiple times with M9 buffer at room temperature to remove residual *E. coli* OP50 42. A subsection of nematodes (grown with or without cadmium supplementation) were suspended in 1 mL of M9 buffer containing 2 μL of acridine orange (0.03 g/2 mL) (Cambridge Bioscience) at room temperature (in the dark) for 1 hour. To remove excess amounts of the dye, worms were subsequently washed two times with M9 buffer and transferred to fresh NGM plates for 1 hour at 20°C. Next, the worms were collected and washed multiple times to remove *E. coli* OP50, then suspended in 1 mL of M9 buffer containing sodium azide (5 mM NaN_3_). Agarose (2%) was added to the immobilised worms at a ratio of 1:1 and the mixture loaded inside the glass capillary.

## 3 RESULTS

### 3.1 Calibration

**Figure 2:**
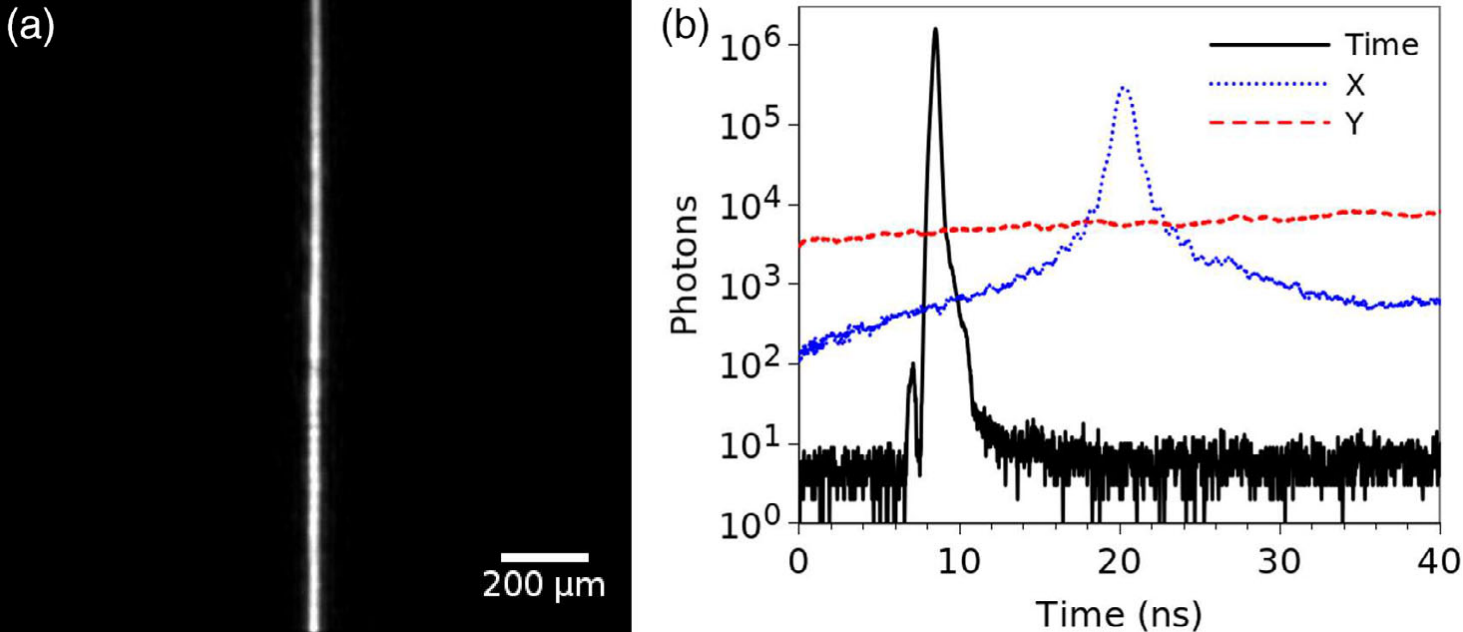
Characterisation of the lightsheet microscope measured by placing a mirror in the sample holder at an angle of 45°. A, Image of the lightsheet. B, Distribution of photons in the time (black), *x* (blue) and *y* (red) channels show the IRF in the time channel and cross‐section of the lightsheet in the *x* channel. The actual measurement period is 100 ns; the edges have been left out for clarity

For the measurement of the lightsheet thickness and the instrument response function (IRF), a mirror was placed in the sample plane at an angle of 45°, and the emission filter replaced with a strong neutral density filter. Data was acquired for ∼5 minutes, with 16.6 million matched photons and count rate of 56.2 kHz. The event acceptance rates for the individual channels were 88.9% for *x*, 86.0% for *y*, and 80.2% for the time channel. Figure 2A shows an image of the reflected lightsheet, and Figure 2B shows the photons collected in the time channel corresponding to the IRF (black line), in the *x*‐channel corresponding to the cross‐section of the lightsheet (blue line), and in *y*‐channel which shows no features (as expected for a cross section of the image along the lightsheet). The IRF measured full width at half maximum (FWHM) is 288 ps, and the lightsheet thickness ∼21 μm.

### 3.2 Fluorescent beads

#### 3.2.1 Slice‐by‐slice imaging

**Figure 3:**
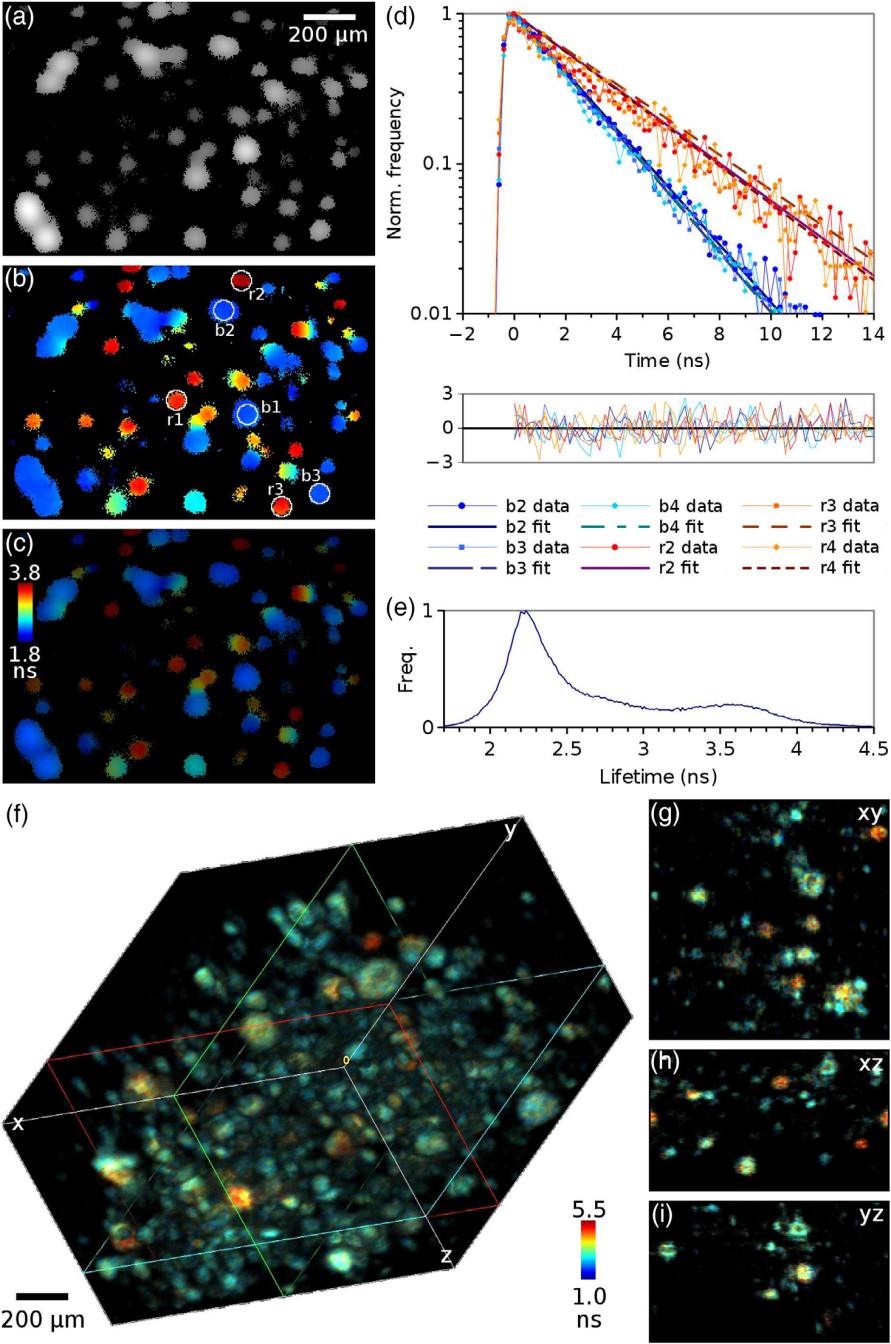
Fluorescent beads imaged with the lightsheet FLIM microscope. A, Intensity; B, lifetime; and C, intensity‐weighted lifetime images of two types of green fluorescent beads. D, Example decays and monoexponential fits to the areas indicated in (B), yielding average lifetimes of 2.21 ± 0.04 and 3.54 ± 0.14 ns for the two types of beads. E, A histogram of lifetimes in the whole stack shows peaks at 2.23 ns and 3.56 ns. In (A,C) gamma has been adjusted for better visualisation of dimmer beads. F, Intensity‐weighted FLIM volume image, obtained by volume sweep imaging, of fluorescent beads and quantum dots, showing lifetime contrast between fluorescent beads (blue/green) that have shorter fluorescence lifetime, and quantum dots (yellow/red) with longer fluorescence lifetime. (G‐I) Cross‐sections of the stack in (F)

The lightsheet FLIM setup was first tested with a sample containing two types of green fluorescent beads which are difficult to distinguish by their emission spectra. A stack was acquired with 10 μm spacing with a total of 44 slices and 2 minutes acquisition time per slice. Intensity and lifetime images of a slice from this stack are shown in Figure 3A‐C. The lifetime images were obtained by fitting a single exponential function to each pixel of the image. Example decays for areas indicated in Figure 3B and monoexponential fits to these decays are shown in Figure 3D, yielding average lifetimes of 2.21 ± 0.04 and 3.54 ± 0.14 ns for the two types of beads. The lifetime histogram of the whole stack (Figure 3E) shows two peaks at 2.23 ns and 3.56 ns, as expected, corresponding to the lifetimes of the two different beads, and, as the density of the beads was quite high such that there is overlap between beads, a distribution of lifetimes between these two values corresponding to pixels that have a contribution from both lifetimes. See Supporting Information for the full stack.

#### 3.2.2 Volume sweep imaging

The lightsheet FLIM microscope can be operated at volume sweep imaging mode, where the sample is moved at constant speed through the lightsheet and data is acquired at a constant stream, and later divided onto slices of required duration. This method was tested with a sample containing a mix of fluorescent beads and quantum dots. A total of 11.3 million photon events were detected in all three (time, x and y) channels during the 6 minutes data collection time, with count rate of 31.6 kHz. The photons were divided into frames of 330 ms, corresponding to a distance of 0.5 μm, with 900 frames in total. The lifetime in each pixel of this *xyzt* data stack was then calculated using the method of moments. Figure 3F shows volume rendering of the intensity‐weighted lifetime image stack, and Figure 3G‐I cross‐sections of this stack in x, y and z dimensions. Although both species emit in green and are difficult to distinguish by emission spectrum or intensity, the lifetimes are different, and the image shows clear lifetime contrast between the beads that have a shorter lifetime and the quantum dots with a longer lifetime.

### 3.3 Cancer cell spheroids

#### 3.3.1 Slice‐by‐slice imaging

**Figure 4:**
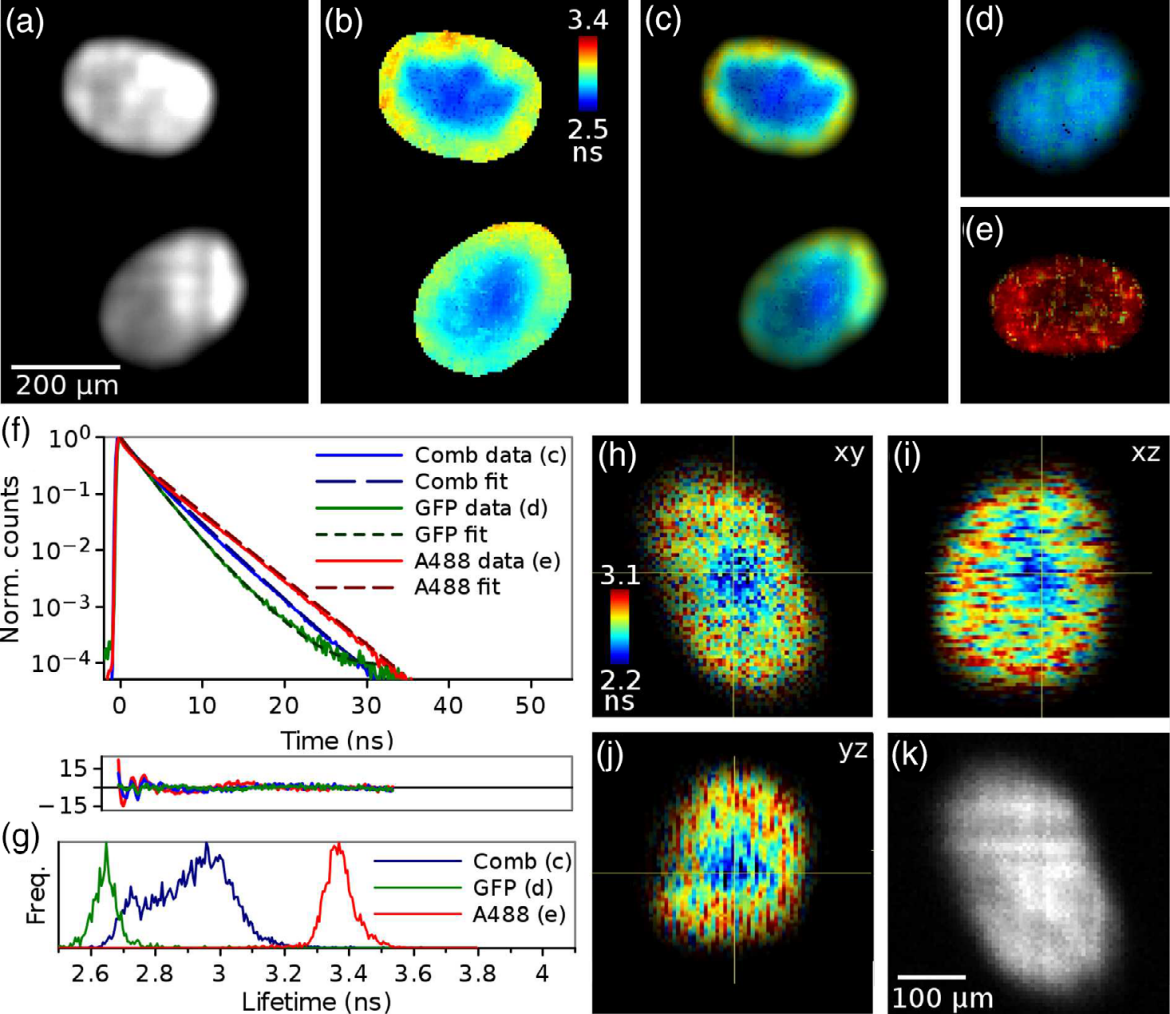
Lightsheet FLIM images of cancer cell spheroids, where the cells were labelled with GFP‐lifeact (shorter lifetime), and the outer surface of the spheroid with Alexa‐488 (longer lifetime). A, Intensity; B, lifetime; and C, intensity‐weighted lifetime images the spheroids with both labels; and D and E, images of control spheroids with only (D) GFP or (E) Alexa‐488. F, Fluorescence decays, biexponential fits to the data and residuals of the fits for images (C‐E), and (G) histograms of the lifetime values in images (C‐E). In (F,G) the data has been averaged over the whole image. H‐K, Volume sweep FLIM images of the spheroids with both GFP and Alexa‐488 labels: H‐J, orthogonal views through the lifetime stack; and H, intensity image of the same *xy* slice

Figure 4 shows TCSPC FLIM images of cancer cell spheroids acquired with the delay line anode detector. The MCF‐7 cells were expressing GFP‐lifeact, labelling the spheroids throughout with GFP, while the outer surface of the spheroids was labelled with Alexa‐488. A total of 31 slices were acquired at 10 μm intervals through the spheroid with data acquisition time 2 minutes per slice. Images of spheroids with both of these labels show nearly uniform intensity where the labels cannot be separated by intensity (Figure 4A), but the distribution of the two labels can be seen as a gradient in the lifetime (Figure 4B,C) with the shorter GFP lifetime contributing mostly in the middle of the spheroid and the longer Alexa‐488 lifetime contribution increasing towards the edges. Control experiments were performed with spheroids that had the GFP (Figure 4D) or Alexa‐488 (Figure 4E) label only.

The fluorescence decays, averaged over the images Figure 4C‐E, are shown in Figure 4F. The average fluorescence lifetimes obtained from biexponential fits to these decays are ${\bar{\tau }}_{\mathrm{GFP}}$=2.39 ns and ${\bar{\tau }}_{A488}$=3.41 ns for the GFP and Alexa‐488 in the control samples with single label (Figure 4D,E), while the average lifetime for the sample with both labels is 2.88 ns. In the lifetime histogram (Figure 4G) the lifetimes from the GFP and Alexa‐488 control images are well separated with narrow peaks at 2.65 and 3.36 ns, respectively. The histogram for the image with both fluorophores is broad, as expected, and shows a minor peak at 2.7 ns corresponding to the spheroid centre with mostly GFP contributing, and then a steady increase towards an average lifetime of 3 ns corresponding to the Alexa‐488 label gradient towards the surface. See Supporting Information for the whole stack.

#### 3.3.2 Volume sweep imaging

The spheroids were also imaged in the volume sweep mode. The 300 μm deep volume was imaged in 5 minutes, and divided into 65 slices with 4.6 μm spacing and data acquisition time of 5 s/slice. A total of 6.56 million photons were collected, corresponding to count rate of 21.5 kHz. Figure 4H‐K shows a slice from this stack, where the intensity image (K) looks uniformly bright but the lifetime image (H‐J) shows the lifetime gradient from the shorter GFP lifetime only in the middle to higher lifetime with increasing Alexa‐488 contribution towards the spheroid surface. Although the method of moments is not as precise as the exponential fitting method and does not allow multicomponent lifetime analysis, the gradient in the lifetime is clearly visible. See Supporting Information for the whole stack.

### 3.4 *C. elegans*

**Figure 5:**
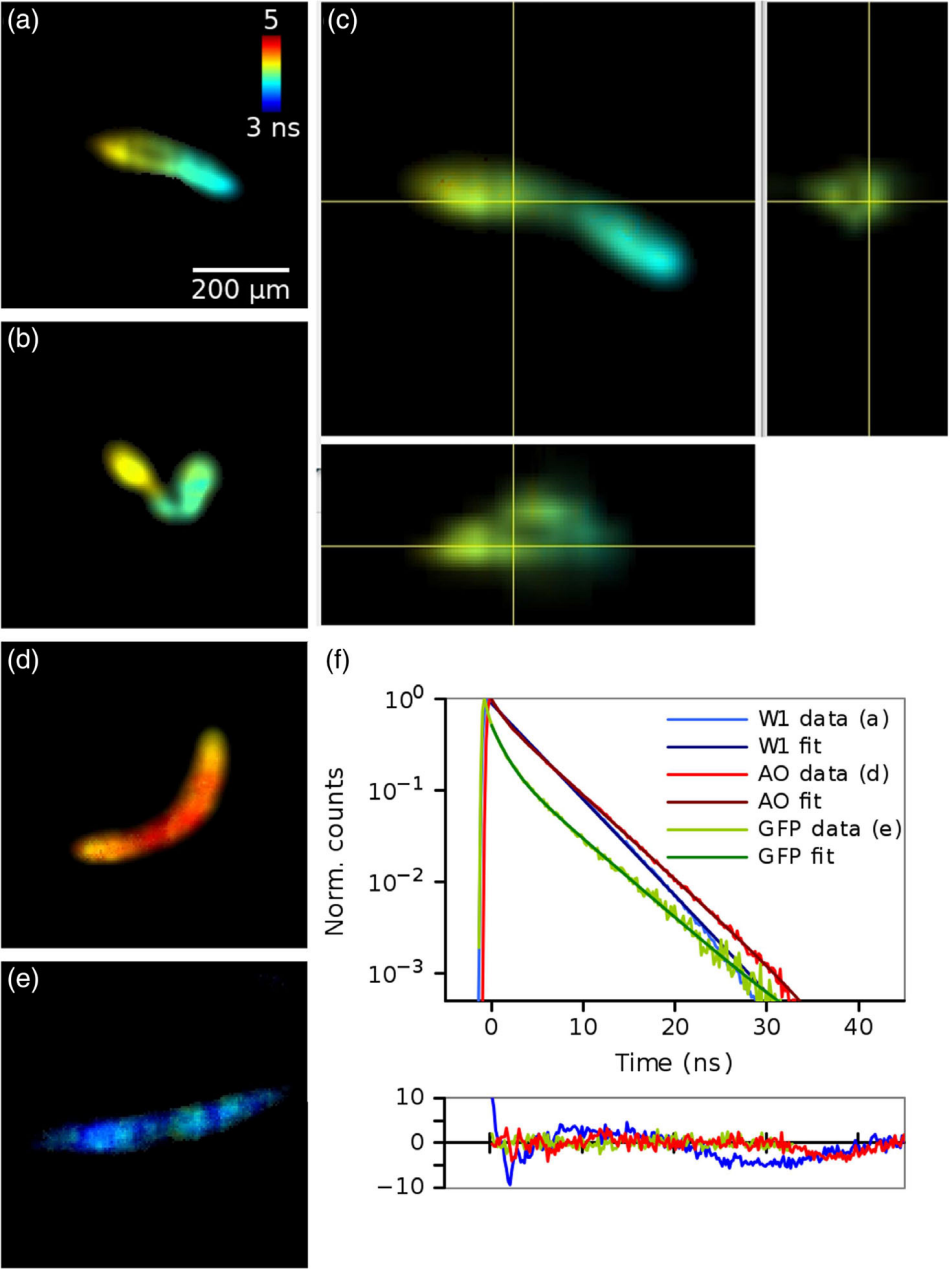
Lightsheet FLIM images of *C. elegans*. Slices from a stack (A,B) and orthogonal views (C) of a stack of *C. elegans* expressing GFP and stained with AO, and images of worms stained only with AO (D) or only expressing GFP (E). F, Time decays, biexponential fits to the data and residuals of the fits for images (A, D, E). Spatial scale in images (B, D, E) and lifetime scale in all images is the same as in (A)

Figure 5 shows TCSPC FLIM images of living *C. elegans* expressing GFP and stained with Acridine Orange (AO). A stack was acquired with the delay line anode detector with 25 μm spacing and 2 minutes acquisition time per slice. The count rates were comparable to the other experiments; for example, a total of 4.5 million photons were collected with count rate of 37.8 kHz for the slice in Figure 5A. Lifetimes were obtained by fitting a double exponential function to each pixel of the image.

*C. elegans* expressing GFP and stained with AO (Figure 5A‐C) have an inhomogeneous distribution of lifetimes throughout the length of the worm with an average lifetime of 4.19 ns. The *C. elegans* containing only AO (Figure 5D) or GFP (Figure 5E) have higher and lower lifetimes of 4.47 and 4.03 ns, respectively. See Supporting Information for example stacks.

## 4 DISCUSSION

We have combined lightsheet microscopy with a TCSPC‐based wide‐field FLIM detector, and illustrated its applications by imaging cancer cell spheroids and *C. elegans*. Lightsheet microscopy enables optical sectioning of thick biological samples embedded in 3D environment while reducing photobleaching of the sample by illuminating only the imaged plane, whereas FLIM provides contrast based on the microenvironment of the probe or proximity of other fluorophores (ie, FRET). As the low excitation volume and low excitation power yield a limited photon count rate, a photon counting approach is indispensable.

Biochemical tools to study protein interactions do not provide spatial or temporal information. Whilst protein interactions can be studied with biochemical assays, they compromise the cell, and, unlike imaging, do not provide dynamic information in real time about protein or cell stimulation upon addition of extracellular factors. Biochemistry is also not possible in some conditions, for example where two different cell types are cultured together.

In this work, the microscope optics were designed to allow a large field of view of ∼1.5 mm diameter to be imaged, with 4 μm pixel size in the sample plane. The pixel size and magnification can be changed according to the sample requirements, for example higher magnification could be used if single cell resolution is required. The image quality and resolution could also be improved by adopting different lightsheet illumination methods, for example illuminating the sample from both sides.

We have applied two different methods for fluorescence lifetime calculation. The conventional fitting methods provide high accuracy and precision that are needed for complex multicomponent lifetime analysis. Picosecond photon timing capabilities of the MCP combined with fast timing electronics allow IRF widths of a few hundred picoseconds to be achieved, ideal for nanosecond fluorescence lifetime measurements. The volume sweep imaging mode, on the other hand, where the lifetime is obtained with the method of moments, allows the acquisition of whole FLIM stacks of large 3D volumes in time scale of a few minutes – ideal for real‐time monitoring of living specimen.

Furthermore, it is possible to improve the overall data acquisition time. In our 40 mm diameter detector the length of the delay line restricts the data collection window to 100 ns, therefore the detector was operated at an excitation repetition rate of 10 MHz. Only one photon per excitation period can be timed, and to avoid photon pile‐up we have chosen to limit the count rate to <1% of the excitation repetition rate, therefore the count rate in these experiments was limited to 10^5^ Hz. It is possible to decrease the size of the detector area from 40 to 25 mm and operate this delay line in faster mode at 50 MHz repetition rate 29, yielding a maximum count rate of 5 × 10^5^ Hz, and shortening the volume sweep mode stack acquisition time from the 5 minutes demonstrated here to just 1 minute. This could be further improved by pile‐up inspection 43 or advanced read‐out architectures 44.

A distinctive advantage of wide‐field TCSPC is extremely low, uniformly distributed illumination intensity. In these experiments, 0.1–1 μW excitation power, adjusted according to the sample brightness, was distributed uniformly over the field of view, yielding a maximum intensity of ∼0.5 to 5 mW/cm^2^. This is significantly lower than 200 mW/cm^2^ reported for gate scanning lightsheet FLIM 10 or the sun's irradiance of the earth, 100 mW/cm^2^.

The applications shown in this manuscript could easily be expanded. Spheroids are often used in cancer studies as a model of real tumour. Here the spheroids were fixed to allow immunolabelling of the spheroid surface, but FLIM can be used in living spheroids for monitoring cell cycle 45, or physiological parameters such as temperature 46 or oxygen concentration 47. We also demonstrated imaging live *C. elegans* with two fluorescent labels; similar technique could be applied for imaging zebrafish, for example. The low excitation power would also allow continuous long‐term imaging 26, 27, 48. Moreover, polarised detection of the fluorescence could also be included 49, 50, to perform time‐resolved fluorescence anisotropy imaging to map viscosity, or binding and cleavage, via the rotational correlation time, or protein interaction or conformational change via homo‐FRET in 3D.

## 5 CONCLUSION

We have combined picosecond wide‐field TCSPC FLIM with lightsheet microscopy. The microwatt illumination intensity required for TCSPC is significantly lower than other wide‐field FLIM methods, and this technique would be ideal for lightsheet microscopy of living organelles, and, for example, measurement of FRET. Besides lightsheet microscopy, this approach is well suited for other specialised illumination microscopy techniques typically employing cameras, such as total internal reflection 30 or supercritical angle fluorescence microscopy 51. It would also enable FLIM for camera‐based super‐resolution fluorescence microscopy techniques relying on single fluorophore localisation, and combine single‐particle tracking measurements with lifetime measurements.

## CONFLICT OF INTEREST

The authors declare no potential conflict of interest.

## Supporting information


**Video S1 beads1‐slowStack.avi** 3D stack of two types of green fluorescent beads acquired slice by slice, 44 slices at 10 μm intervals with data acquisition time 2 minutes per slice. Lifetime scale 1.7–4.2 ns, left = intensity, right = lifetime. The video shows a fly‐though the focus stack slice by slice.

**Video S2 spheroid1‐slowStack.avi** 3D stack of a cancer cell spheroid acquired slice by slice, 31 slices at 10 μm intervals with data acquisition time 2 minutes per slice. Lifetime scale 2.5 to 3.4 ns. The cells were labelled with GFP‐lifeact (shorter lifetime), and the outer surface of the spheroid with Alexa‐488 (longer lifetime). The video shows brightest point projection along y‐axis rotated through 360 degrees, and a fly‐though the focus stack slice by slice.

**Video S3 spheroid2‐fastStack.avi** Fast FLIM stack of a cancer cell spheroid, left = intensity, right = lifetime. 65 slices at 4.6 μm intervals with data acquisition time 5 seconds per slice. Lifetime scale 2.2–3.1 ns. The cells were labelled with GFP‐lifeact (shorter lifetime), and the outer surface of the spheroid with Alexa‐488 (longer lifetime). The video shows a fly‐though the focus stack slice by slice.

**Video S4 worm‐XX.avi** 3D stacks of *C. elegans* acquired slice by slice. “AO” and “GFP” are controls with one label only, “resXX” have both labels. Lifetime scale 3–5 ns. The videos show a fly‐though the focus stack slice by slice upwards and downwards, brightest point projection along y‐axis rotated through 360 degrees, and brightest point projection along x‐axis rotated through 360 degrees.
